# Triglyceride-glucose index, plasma metabolome and aortic stenosis: a Mendelian randomization analysis

**DOI:** 10.1186/s13019-026-03976-3

**Published:** 2026-04-06

**Authors:** Zhisheng Yan, Yikang Wang, Xiaomei Dai, Qing Chang

**Affiliations:** 1 Department of Cardiovascular Surgery, The Affiliated Hospital of Qingdao University, Qingdao University, No.16 Jiangsu Road, Qingdao, 266003 China; 2 School of Medicine, South China University of Technology, Guangzhou, China; 3 Department of Emergency Medicine, Guangdong Provincial People’s Hospital, Guangdong Academy of Medical Sciences, Southern Medical University, Guangzhou, Guangdong China

**Keywords:** Mendelian randomization, Triglyceride-glucose index, Plasma metabolites, Aortic stenosis, FinnGen

## Abstract

**Background and Aims:**

The association between the Triglyceride-Glucose index, an emerging marker of insulin resistance, and the risk of aortic stenosis remains unclear. The influence of Triglyceride-Glucose index on aortic stenosis may be mediated through plasma metabolites. To investigate the potential causal relationships between the Triglyceride-Glucose index, plasma metabolites, and aortic stenosis, we employed a two-sample Mendelian randomization approach.

**Methods:**

We conducted univariable Mendelian randomization analyses to explore the associations between (i) the Triglyceride-Glucose index and aortic stenosis; (ii) plasma metabolites and aortic stenosis; and (iii) the Triglyceride-Glucose index and plasma metabolites. For significant associations identified through Mendelian randomization, a two-step MR analysis was employed to evaluate mediation effects.

**Results:**

The genetically predicted Triglyceride-Glucose index is positively associated with aortic stenosis (OR 1.74, 95% confidence interval 1.41, 2.16). Following adjustments for multiple testing, there were 15 notable associations between genetically predicted metabolites and aortic stenosis. Additional analyses estimated the indirect effects of the TyG index on AS through these metabolites. Notably, the mediation effect of various metabolites was quantified: 1-arachidonoyl-gpc (20:4n6) exhibited a mediation effect of 0.06 (95% CI: 0.02 to 0.11, *P* = 4.38E-03); 1-stearoyl-2-arachidonoyl-gpc (18:0/20:4) had a mediation effect of 0.08 (95% CI: 0.03 to 0.12, *P* = 6.60E-04); 1-stearoyl-2-arachidonoyl-gpe (18:0/20:4) showed an effect of 0.12 (95% CI: 0.06 to 0.17, *P* = 6.58E-05); 1-palmitoyl-2-arachidonoyl-gpe (16:0/20:4) demonstrated a mediation effect of 0.07 (95% CI: 0.03 to 0.11, *P* = 8.75E-04); 1-palmitoyl-2-docosahexaenoyl-gpe (16:0/22:6) showed an effect of 0.09 (95% CI: 0.03 to 0.14, *P* = 1.26E-03); and 1-oleoyl-2-arachidonoyl-gpe (18:1/20:4) had an effect of 0.08 (95% CI: 0.03 to 0.13, *P* = 5.38E-04).

**Conclusion:**

The genetically predicted Triglyceride-Glucose index is positively associated with the risk of aortic stenosis, and this relationship is partially mediated by specific metabolites.

**Supplementary Information:**

The online version contains supplementary material available at 10.1186/s13019-026-03976-3.

## Introduction

Calcific aortic stenosis (AS) is the most common valvular heart disease in high-income populations [1], and it is projected to more than double by 2040 [2]. However, in regions where access to cardiac surgery and interventional cardiology is limited, the prevalence of AS will disproportionately increase. For symptomatic AS patients who are unable to undergo valve replacement surgery, the average survival rates are less than two years [3], with three-quarters progressing to heart failure, undergoing surgery, or facing mortality within five years [4]. Currently, there are no approved pharmacological treatments for calcific aortic valve disease (CAVD). Although aortic valve replacement surgery is effective for severe AS cases, there are currently no methods available to prevent disease progression to the point of requiring surgery.

Insulin resistance is increasingly recognized as a systemic metabolic disorder that extends beyond impaired glucose homeostasis and plays an important role in cardiovascular remodeling. The triglyceride–glucose (TyG) index is a widely used surrogate marker of insulin resistance and has been validated against the hyper insulinemic–euglycemic clamp [5, 6]. Accumulating evidence suggests that metabolic disturbances closely related to insulin resistance—such as chronic low-grade inflammation, dyslipidemia, oxidative stress, and endothelial dysfunction—may contribute to the pathogenesis of calcific aortic valve disease. These processes can promote inflammatory activation within the aortic valve and facilitate osteogenic differentiation of valvular interstitial cells, ultimately leading to valvular calcification and stenosis [7–9]. Previous studies have documented a positive correlation between the TyG index and the risk of various metabolic and atherosclerotic cardiovascular diseases, including heart failure [10–12]. However, research exploring the link between the TyG index and the risk of AS is still limited, and the causal relationship remains unconfirmed.

Metabolites are small molecules that serve as intermediates or products in metabolic pathways [13, 14]. In comparison to other omics disciplines, such as proteomics or genomics, metabolomics offers a more comprehensive reflection of the dynamic changes in the body’s phenotype [15, 16]. Through the study of metabolites and their associated metabolic pathways, we can enhance our understanding of how abnormal metabolism contributes to the onset and progression of diseases. Consequently, alterations in the levels of small metabolites can be utilized as diagnostic and prognostic biomarkers, as well as therapeutic targets [17].

Randomized controlled trials (RCTs) are considered the gold standard method for evaluating the effectiveness of intervention measures due to their scientific rigor and robust internal validity. However, the high costs and time-consuming nature of RCTs make strict implementation challenging. MR provides a valuable approach for assessing assumed causal relationships between plasma metabolites and aortic valve calcification [18–20]. It effectively addresses common limitations encountered in classical epidemiological studies, such as confounding and reverse causality. By utilizing genetic variations that are specifically associated with the presumed exposure as instrumental variables, MR allows for the inference of the causal impact of the exposure on the outcome [20, 21]. At the time of conception, the random assortment of allele ensures that the distribution of genetic variations related to a specific exposure is largely independent of factors that may confound the exposure-outcome relationship in traditional observational analysis [22]. Therefore, in comparison to classical epidemiological studies, MR estimation is less influenced by environmental confounding factors and provides more reliable insights into the causal relationship between risk factors and disease outcomes [18]. Additionally, as an individual’s genotype is determined at conception and remains unaffected by subsequent disease outcomes, the direction of the causal relationship consistently points from genetic variation to the trait of interest, effectively eliminating the possibility of reverse causality [23]. Consequently, since genetic variants are measured after the onset of the disease outcome, MR provides distinct advantages in obtaining reliable causal inferences in retrospective settings [24].

In this study, our research objectives were as follows: (i) to investigate the association between TyG index and AS using MR; (ii) to evaluate whether the TyG index has a causal effect on plasma metabolites; (iii) to assess whether plasma metabolites causally influence the risk of AS; and (iv) to use the two-step MR analysis which can investigate mediating relationships, explore whether the pathogenic effect of the TyG index on the risk of AS is mediated by changes in plasma metabolites.

## Materials and methods

### Data sources

**Fig. 1:**
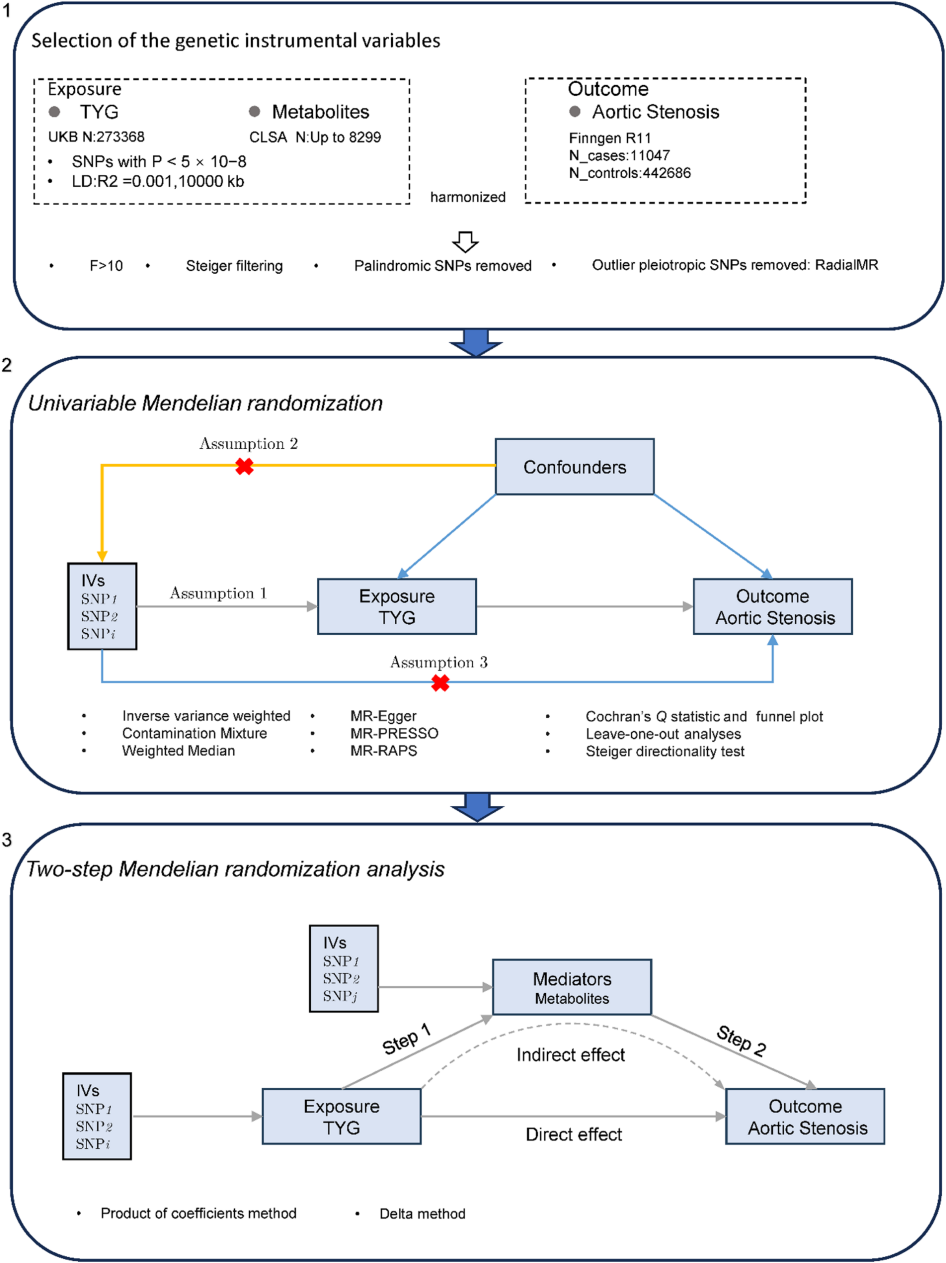
Research Process Flowchart

This study is based on the Two-Sample MR analysis method, where the effects of genetic instruments on exposure and outcomes come from different datasets. All data sources were limited to individuals of European ancestry to minimize potential bias arising from population stratification. These data come from different countries. Furthermore, there were no overlapping samples in the analyses of the TyG index and plasma metabolites associated with AS, with little or no overlap. Figure 1 summarizes the research process of this study.

### Instrumental variables of TyG index

Genetic variants associated with the TyG index were derived from previous genome-wide association studies (GWAS) based on the UK Biobank cohort [25]. Concisely, the GWAS data encompassed 273,368 participants aged between 40 and 69 years, none of whom had diabetes or lipid metabolism disorders. Using linear regression adjusted for age, sex, and the top five genetic principal components to mitigate population stratification, effects related to instrumental SNPs achieved a genome-wide significance level of *P* < 5 × 10^-8. To ensure the independence of these SNPs, they were pruned based on linkage disequilibrium, targeting an R^2 threshold of less than 0.01. Lastly, SNPs significantly associated with both triglycerides (TG) and glucose (GLU), as well as non-lipid and non-glycemic factors such as systolic blood pressure (SBP), diastolic blood pressure (DBP), and body mass index (BMI), were excluded to control for potential horizontal pleiotropy. The TyG index was calculated using the following formula, ln [TG (mg/dl) × FBG (mg/dl)/2].

### GWAS summary statistics of AS

We acquired summary statistics of GWASs associated with AS from the FinnGen database, which included 12,418 cases and 487,930 controls (R12 version) [26]. The FinnGen database defines AS using the International Classification of Diseases (ICD) codes, including I35.0 and I35.2 from ICD-10.

### Potential mediators

This study utilized recent and comprehensive summary statistics from Genome-Wide Association Studies (GWASs) related to plasma metabolites. The dataset included 1,091 plasma metabolites and 309 metabolite ratios, obtained from the study by Chen et al. [27]. The metabolomics investigation focused on 8,299 unrelated European participants who were part of the Canadian Longitudinal Study on Aging (CLSA).

### Selection of the genetic instrumental variables and data harmonization

In our study, we utilized a genome-wide significance threshold (*P* < 5 × 10^-8) to identify Single Nucleotide Polymorphisms (SNPs) suitable as instrumental variables for each exposure. We then applied local linkage disequilibrium pruning to these selected instruments using the “ieugwasr” package, setting a threshold of r^2 < 0.001 to ensure approximate independence across regions exceeding 10,000 kilobase pairs. The European super-population from the 1000 Genomes Project phase 3 was chosen as the reference panel, and this dataset only included biallelic SNPs with a minimum allele frequency (MAF) higher than 0.01. Following this, we harmonized the GWAS datasets for exposure and outcome to ensure that genetic variant association estimates were aligned with the impact of the same allele. By leveraging allele frequency information, we inferred the orientation of alleles in both the exposure and outcome GWAS datasets. We excluded palindromic SNPs with a minor allele frequency exceeding 0.42 [28]. Outlier pleiotropic SNPs were removed following a heterogeneity test, employing modified Q statistics, conducted via RadialMR with a P-value threshold established at 0.05 [29]. *F*-statistic is a measure of instrument strength, and its calculation formula is F = R^2^(N - k − 1) / k (1 - R^2^) [30, 31]. In this formula, N represents the sample exposure size, k represents the number of instruments, and R^2^ represents the degree of exposure explained by the instruments, which is the proportion of phenotypic variance explanation. When the *F*-statistic is greater than or equal to 10, it indicates a relatively weak impact of weak instrument variable bias in MR analysis [32]. R^2^ is obtained through approximate calculation, with the formula R^2^ = β^2^ /(β2 + N×(se(β))^2^ [33]. In this formula, where β represents the effect size estimate, N denotes the sample size, and se(β) indicates the standard error of the effect size for the genetic variant. To orient the direction of causality between the exposure and the outcome, we utilized the Steiger directionality test, an extension of the two-sample Mendelian Randomization [34]. This test identifies variants with a stronger association with the outcome than with the exposure. Based on evidence from the Steiger test revealing that certain genetic instruments had a stronger association with the outcome, we excluded these variants.

In selecting instrumental variables for metabolites, we excluded those with fewer than four SNPs to fulfill the minimum criteria of various MR sensitivity analysis techniques. Ultimately, 266 distinct metabolites were incorporated into the study.

For the statistical power calculations of each MR analysis, we applied the method described by Brion et al. and performed the calculations using the R code provided in the *mRnd* repository (https://github.com/kn3in/mRnd) [35]. 

### Statistical analyses

#### Univariable Mendelian randomization

In this study, we utilized TyG index-associated variants as the exposure and aortic valve stenosis as the outcome employing the MR method. The three foundational assumptions essential for valid outcomes in a MR analysis are as follows. Firstly, the genetic variant employed as an instrumental variable for the risk factor must: (i) reliably associate with the risk factor under examination, thereby meeting the relevance criterion; (ii) remain unassociated with any known or potential confounding variables, thereby aligning with the independence criterion; and (iii) have no direct effect on the outcome, only potentially an indirect effect via the exposure (exclusion restriction). Following Burgess’s recommendation, we employed the inverse-variance weighted with multiplicative random-effects model as the primary analysis method [24, 36]. To investigate the association between metabolites and AS, and to streamline the organization of results for further analysis, we utilized the Benjamini-Hochberg method for adjusting multiple comparisons.

### Sensitivity analyses

For each MR analysis, besides the Inverse Variance Weighted (IVW) method, we utilize five additional methods to assess the robustness of findings with respect to potential MR assumption violations. These methods include MR-RAPS [37], MR-Weighted Median [38], the Contamination Mixture method [39], MR-Egger [40], and the MR pleiotropy residual sum and outlier (MR-PRESSO) [41], These robust methods were selected because they each provide a valid estimate of the causal effect of the exposure on the outcome under varying assumptions [24].

In addition to these robust methods, we conducted a series of additional sensitivity analyses. Firstly, for two or more available instrumental variables, we performed heterogeneity analysis and horizontal pleiotropy analysis using the Cochran’s Q statistic and MR-Egger intercept test [42, 43]. Secondly, we generated funnel plots that depict the relationship between instrumental variable precision and their estimated values [44]. The funnel plots exhibit symmetry with the IVW estimates, with more accurate estimates showing lower variability. The presence of asymmetry in the funnel plots indicates directional pleiotropy and bias in the overall estimation of causality [44]. Thirdly, we conducted leave-one-out sensitivity analyses by systematically excluding individual SNPs to evaluate the potential bias of causal effects due to horizontal pleiotropy [45]. In addition, as a supplementary sensitivity analysis, we excluded SNPs located at the *FADS* locus from the MR analyses of the TyG index and the significant metabolites, as variants in this region exhibit pronounced pleiotropy, being associated with multiple metabolites and atherosclerosis (AS). We then compared the MR estimates before and after removal of these pleiotropic SNPs to assess the robustness of the results. Finally, we employed reverse Mendelian randomization to test for potential reverse causality.

### Mediation analysis

In this two-step MR analysis, genetic instruments associated with the TyG index were first used to estimate its causal impact on potential mediators. Following this, genetic instruments for the identified mediators assessed their causal effects on AS. If the TyG index influenced a mediator, which in turn affected AS, the “product of coefficients” method was employed to determine the TyG index’s indirect effect on AS risk via each mediator [46]. Standard errors for these indirect effects were computed using the delta method [47].

### Ethical approval

This study relies on a secondary analysis of publicly accessible GWAS data. The ethical approval can be located within the original article.

### Packages availability

For the statistical analysis, we used the “TwoSampleMR” package (version 0.6.1) (https://github.com/MRCIEU/TwoSampleMR). LD clumping was performed using the “ieugwas” package (version 0.4.10) (https://github.com/MRCIEU/ieugwasr) and plink 1.90. The European reference panel is available at (http://fileserve.mrcieu.ac.uk/ld/1kg.v3.tgz). Sensitivity analysis involved the use of the “MR-RAPS” package (version 0.4.10) (https://github.com/qingyuanzhao/mr.raps). The subsequent analysis was conducted using the “MendelianRandomization” package (version 0.10.0) (https://github.com/cran/MendelianRandomization), “MRPRESSO” package (version 1.0). All analyses were performed using R (Version 4.3.1, R Foundation for Statistical Computing, Vienna, Austria. URL https://www.R-project.org/.).

## Results

### Robustness of genetic instruments

This study performed MR analysis on 266 unique plasma metabolites. The F-statistics for the genetic instruments linked to the TyG index and each metabolite consistently exceeded 10, indicating the instruments used in the research are unlikely to be weak.

### Positive association of genetically predicted TyG index with increased risk of AS

**Fig. 2:**
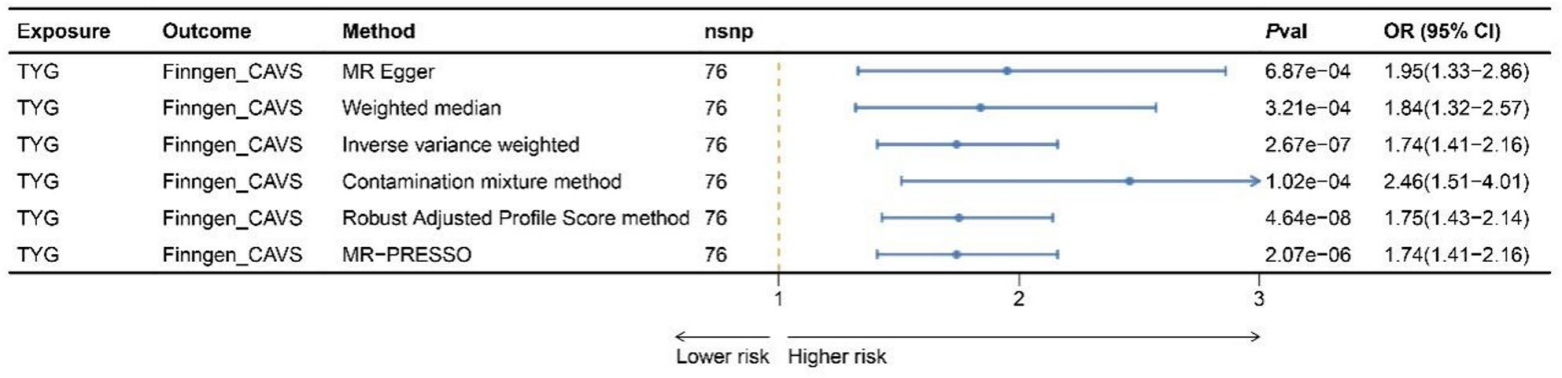
MR results for the relationship between TyG index and aortic stenosis

**Fig. 3:**
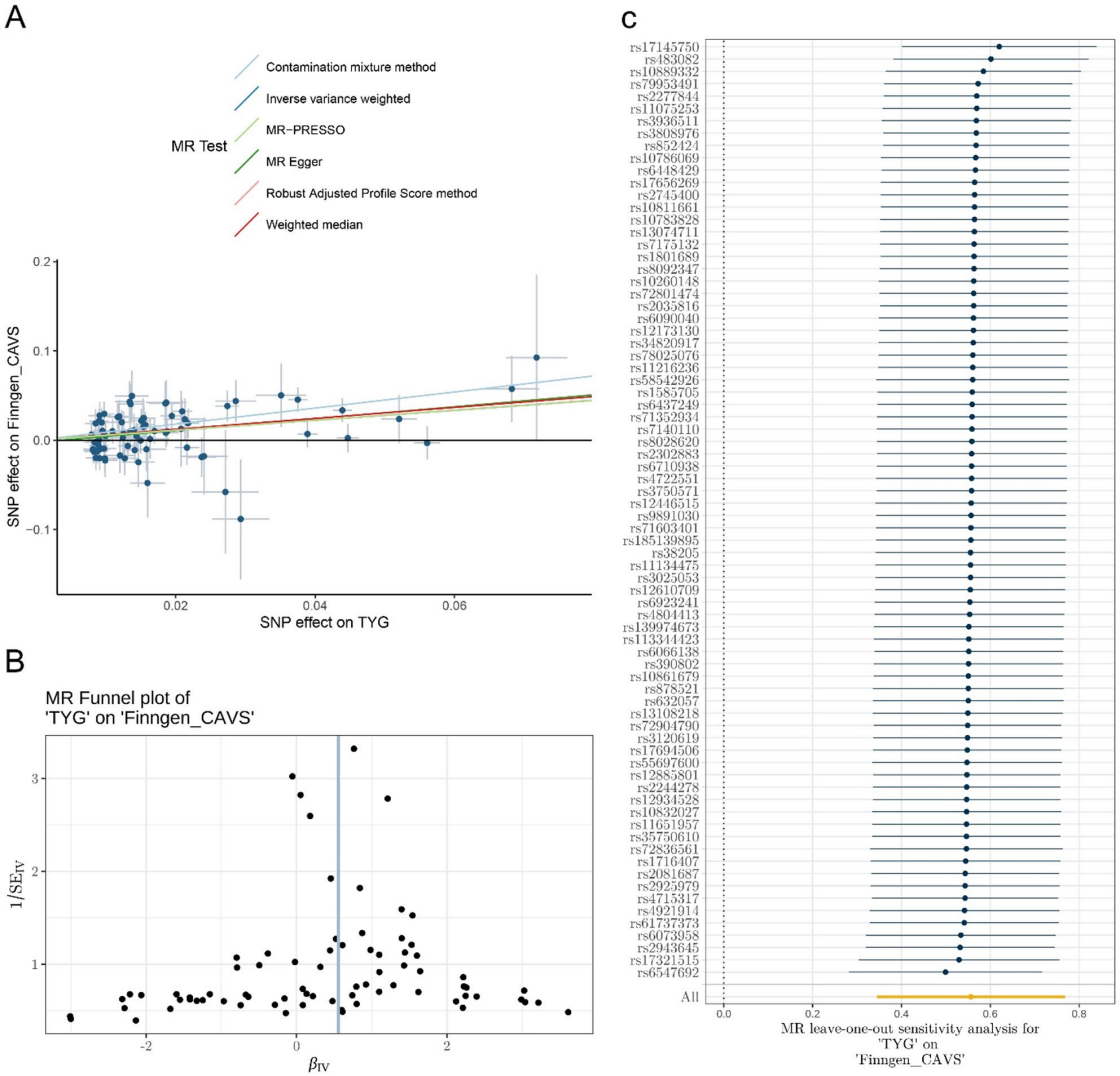
MR plots for the relationship between TYG index and AS. Panel **A** displays a scatter plot of SNP effects on TYG index and AS, with each line’s slope representing the estimated MR effect per method. Panel **B** shows a funnel plot, and Panel **C** presents a leave-one-out plot

Substantial evidence indicates a positive association between the genetically proxied TyG index and AS using various MR methods. The main IVW analysis revealed that each SD increase in the genetically predicted TyG index was associated with a 70% increase in the odds of AS (OR = 1.70, 95% CI: 1.41–2.16). Similar estimations were provided by the MR-RAPS and MR-PRESSO methods, whereas the Weighted Median, MR-Egger, and contamination mixture methods produced larger direct estimates (Fig. 2). Figure 3A presents scatter plots illustrating the association between the genetically proxied TyG index and the risk of AS for the selected instruments, with each regression analysis represented by lines of different colors. Cochran’s Q statistic revealed no significant heterogeneity in the variant-specific causal estimates beyond what would be expected by chance (Cochran’s Q statistic = 84.66, P-value = 0.21). Additionally, the MR-Egger intercept test showed no evidence of directional pleiotropy (P-value = 0.50), the funnel plot indicated no departure from symmetry (Fig. 3B), and the leave-one-out plot displayed no distortions (Fig. 3C and Supplementary Table 4). As a further sensitivity analysis, we redefined the genetic instruments for the TyG index by excluding SNPs at the *FADS* locus. The associations were largely unchanged, and no substantial heterogeneity or horizontal pleiotropy was observed (Supplementary Tables 26–28).

### Association of genetically predicted metabolites with AS

**Fig. 4:**
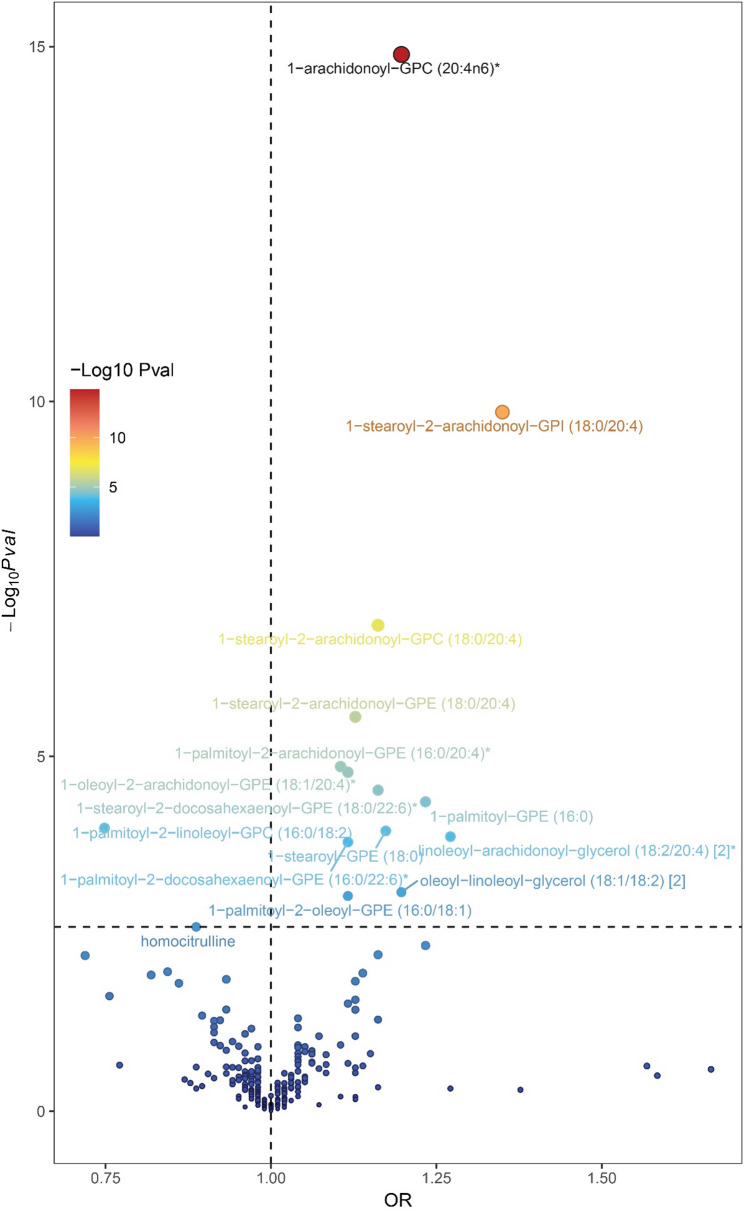
MR results for plasma metabolites and the risk of AS. Volcano plots display the results of univariable Mendelian Randomization analyses for 266 plasma metabolites associated with the risk of Aortic Stenosis. These analyses utilized either the Wald ratio or the inverse variance-weighted method. A horizontal black line indicates the Benjamin-Hochberg (BH) adjusted significance threshold

**Fig. 5:**
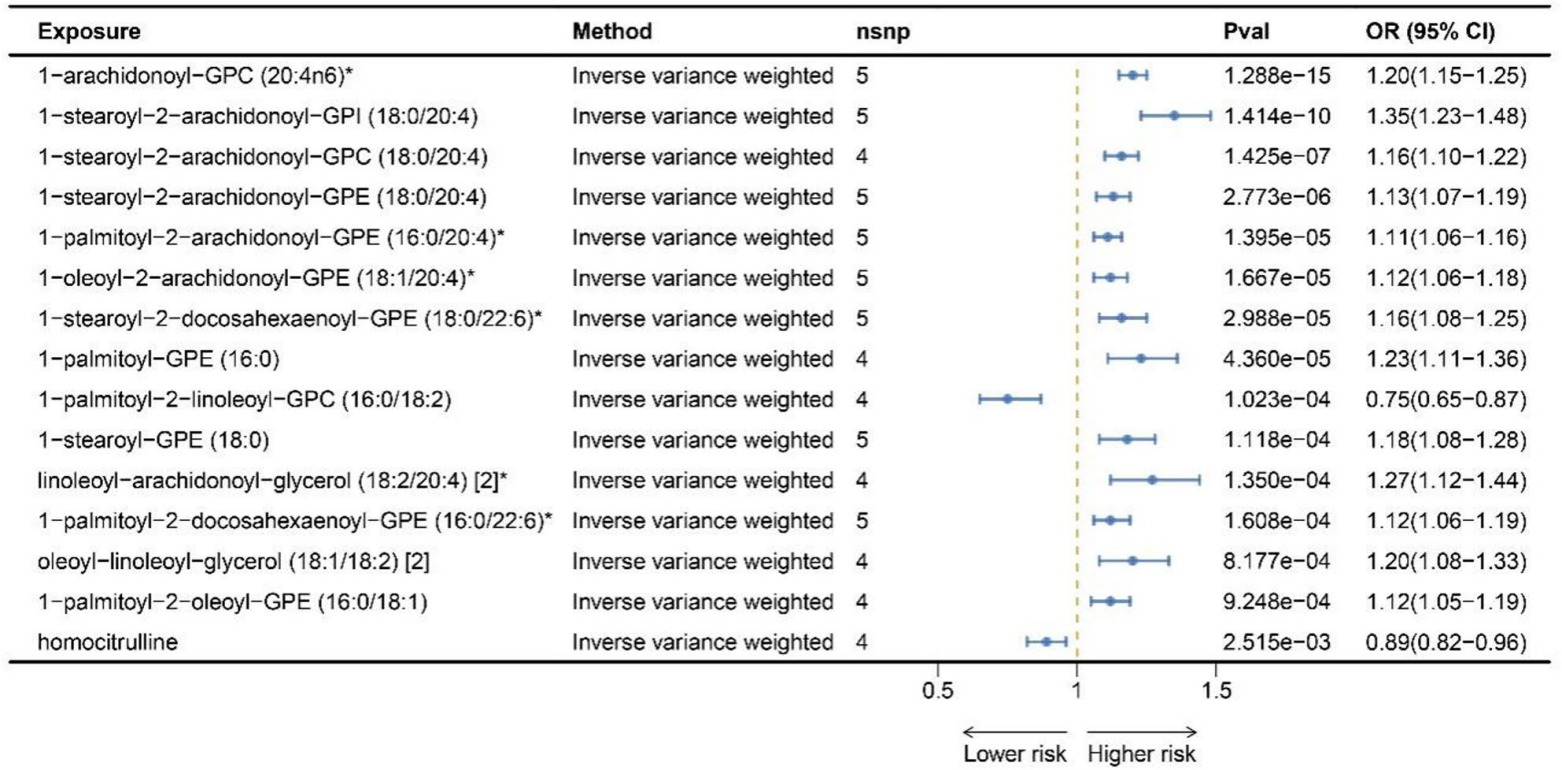
MR analyses identified 15 unique plasma metabolites significantly associated with Aortic Stenosis following Benjamin-Hochberg correction

Using the main IVW method to set the threshold for multiple comparison adjustments, MR analysis identified associations between fifteen unique metabolites and AS, including 1-arachidonoyl-GPC (20:4n6, OR = 1.20, 95% CI: 1.16–1.24, P_FDR_ = 3.43 × 10⁻¹³), 1-stearoyl-2-arachidonoyl-GPI (18:0/20:4, OR = 1.35, 95% CI: 1.23–1.48, P_FDR_ = 1.88 × 10⁻⁸), 1-stearoyl-2-arachidonoyl-GPC (18:0/20:4, OR = 1.16, 95% CI: 1.10–1.22, P_FDR_ = 1.26 × 10⁻⁵), 1-stearoyl-2-arachidonoyl-GPE (18:0/20:4, OR = 1.13, 95% CI: 1.07–1.19, P_FDR_ = 1.84 × 10⁻⁴), 1-palmitoyl-2-arachidonoyl-GPE (16:0/20:4, OR = 1.11, 95% CI: 1.06–1.16, P_FDR_ = 7.39 × 10⁻⁴), 1-oleoyl-2-arachidonoyl-GPE (18:1/20:4, OR = 1.12, 95% CI: 1.06–1.18, P_FDR_ = 7.39 × 10⁻⁴), 1-stearoyl-2-docosahexaenoyl-GPE (18:0/22:6, OR = 1.16, 95% CI: 1.08–1.25, P_FDR_ = 1.14 × 10⁻³), 1-palmitoyl-GPE (16:0, OR = 1.23, 95% CI: 1.11–1.36, P_FDR_ = 1.45 × 10⁻³), 1-stearoyl-GPE (18:0, OR = 1.18, 95% CI: 1.08–1.28, P_FDR_ = 2.97 × 10⁻³), 1-palmitoyl-2-linoleoyl-GPC (16:0/18:2, OR = 0.75, 95% CI: 0.67–0.87, P_FDR_ = 2.97 × 10⁻³), linoleoyl-arachidonoyl-glycerol (18:2/20:4, OR = 1.27, 95% CI: 1.12–1.44, P_FDR_ = 3.27 × 10⁻³), 1-palmitoyl-2-docosahexaenoyl-GPE (16:0/22:6, OR = 1.12, 95% CI: 1.06–1.19, P_FDR_ = 3.56 × 10⁻³), oleoyl-linoleoyl-glycerol (18:1/18:2, OR = 1.20, 95% CI: 1.08–1.33, P_FDR_ = 1.67 × 10⁻²), 1-palmitoyl-2-oleoyl-GPE (16:0/18:1, OR = 1.12, 95% CI: 1.05–1.19, P_FDR_ = 1.76 × 10⁻²), and homocitrulline (OR = 0.89, 95% CI: 0.82–0.96, P_FDR_ = 4.46 × 10⁻²), as detailed in Supplementary Tables 5 and Figs. 4 and 5. Other robust MR methods yielded approximate estimates as well (Supplementary Table 6). Although the MR-Egger confidence intervals for certain metabolite-AS pairs encompassed the null value, the point estimates consistently aligned in the same direction, and all remaining robust methods corroborated the IVW analysis (Supplementary Table 6). For these fifteen metabolite-AS pairs, all of Cochran’s Q statistics revealed no significant heterogeneity (Supplementary Table 7), and the MR-Egger intercept test showed no evidence of directional pleiotropy (Supplementary Table 8). Additionally, the leave-one-out analysis displayed no distortions (Supplementary Table 9), and all pairs passed the Steiger directionality test (Supplementary Table 10). For metabolites that showed significant associations in our primary MR analysis, we performed reverse Mendelian randomization to evaluate potential reverse causality. When aortic stenosis (AS) was considered as the exposure and these metabolites as the outcomes, all P-values were > 0.05 (Supplementary Table 19). Additionally, no evidence of horizontal pleiotropy or heterogeneity was detected. These results do not provide support for a causal effect of AS on the metabolites (Supplementary Table 20). Similarly, we performed an additional sensitivity analysis in which SNPs located near the *FADS* locus were excluded from the genetic instruments for each metabolite. After exclusion of these variants, four metabolites were no longer significantly associated with AS, including 1-stearoyl-2-arachidonoyl-GPC (18:0/20:4), 1-stearoyl-2-arachidonoyl-GPI (18:0/20:4), linoleoyl-arachidonoyl-glycerol (18:2/20:4), and 1-palmitoyl-2-linoleoyl-GPC (16:0/18:2) (Supplementary Tables 23–25).

### Association of genetically predicted TyG index with metabolites

**Fig. 6:**
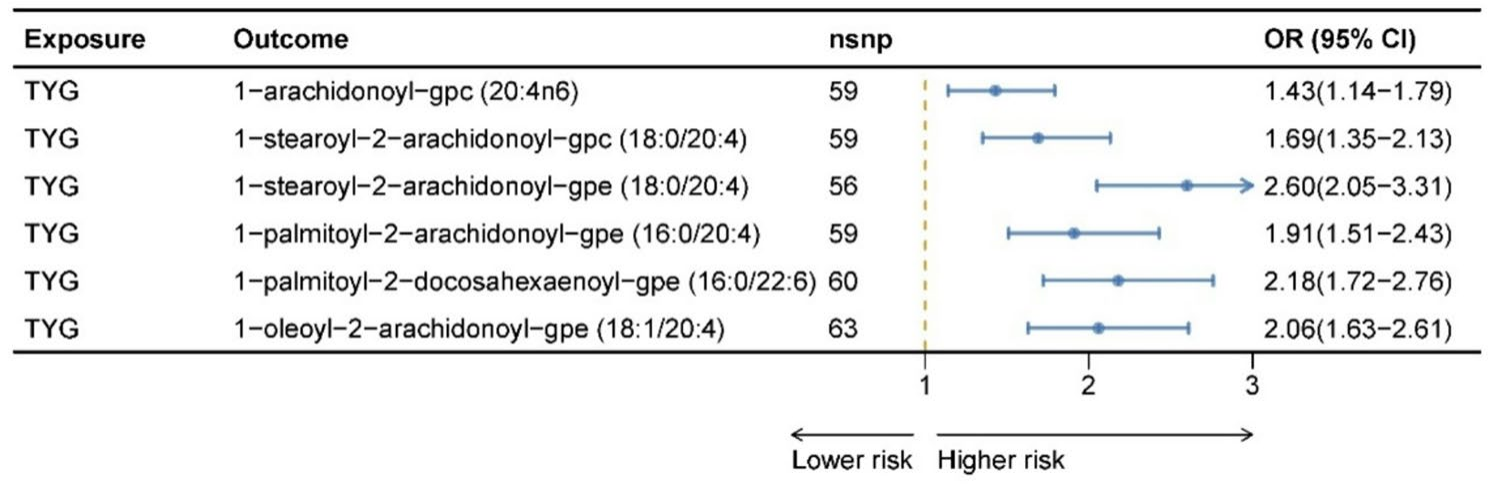
MR results for the relationship between predicted TyG index and plasma metabolites

We examined the associations between the TyG index and fifteen mentioned metabolites. Upon implementing multiple testing corrections, thirteen tyg-metabolite pairs met the predefined significance criteria. Despite the absence of significant heterogeneity in these metabolite-AS pairs as evidenced by Cochran’s Q statistics, the MR-Egger intercept test identified directional pleiotropy in compounds such as 1-stearoyl-gpe (18:0), oleoyl-linoleoyl-glycerol (18:1/18:2) [2], 1-palmitoyl-gpe (16:0), 1-stearoyl-2-arachidonoyl-gpi (18:0/20:4), linoleoyl-arachidonoyl-glycerol (18:2/20:4) [2], 1-stearoyl-2-docosahexaenoyl-gpe (18:0/22:6), and 1-palmitoyl-2-oleoyl-gpe (16:0/18:1) (Supplementary Tables 13 and 14). Consequently, these pleiotropic metabolites were removed from further analysis. All TyG index–metabolite pairs passed the Steiger directionality test (Supplementary Table 16). Ultimately, six notable metabolites were identified, including 1-arachidonoyl-GPC (20:4n6, OR = 1.43, 95% CI: 1.14–1.79, P_FDR_ = 2.44 × 10⁻³), 1-stearoyl-2-arachidonoyl-GPC (18:0/20:4, OR = 1.69, 95% CI: 1.35–2.13, P_FDR_ = 6.79 × 10⁻⁶), 1-stearoyl-2-arachidonoyl-GPE (18:0/20:4, OR = 2.60, 95% CI: 2.05–3.31, P_FDR_ = 3.53 × 10⁻¹⁴), 1-palmitoyl-2-arachidonoyl-GPE (16:0/20:4, OR = 1.91, 95% CI: 1.55–2.43, P_FDR_ = 1.47 × 10⁻⁷), 1-palmitoyl-2-docosahexaenoyl-GPE (16:0/22:6, OR = 2.18, 95% CI: 1.72–2.76, P_FDR_ = 2.32 × 10⁻¹⁰), and 1-oleoyl-2-arachidonoyl-GPE (18:1/20:4, OR = 2.06, 95% CI: 1.63–2.61, P_FDR_ = 3.46 × 10⁻⁹), with corroborative results from alternative sensitivity analyses consistent with those derived via the IVW method (Supplementary Tables 12 and Fig. 6).

### Mediation analysis

We analyzed the indirect effects of the TyG index on AS through six metabolites and documented various significant mediation effects (Table 1). Notably, the mediation effect of 1-arachidonoyl-gpc (20:4n6) was estimated at 0.06 (95% CI 0.02 to 0.11, *P* = 4.38E-03), accounting for 11.56% of the effect. Similarly, the effect of 1-stearoyl-2-arachidonoyl-gpc (18:0/20:4) was 0.08 (95% CI 0.03 to 0.12, *P* = 6.60E-04), representing 13.89% of the total effect. Furthermore, 1-stearoyl-2-arachidonoyl-gpe (18:0/20:4) exhibited a mediation effect of 0.12 (95% CI 0.06 to 0.17, *P* = 6.58E-05), contributing to 21.09%. The 1-palmitoyl-2-arachidonoyl-gpe (16:0/20:4) showed a mediation of 0.07 (95% CI 0.03 to 0.11, *P* = 8.75E-04), corresponding to 12.24%. Additionally, the mediation effect of 1-palmitoyl-2-docosahexaenoyl-gpe (16:0/22:6) was 0.09 (95% CI 0.03 to 0.14, *P* = 1.26E-03), impacting 15.83%, while 1-oleoyl-2-arachidonoyl-gpe (18:1/20:4) had an effect size of 0.08 (95% CI 0.03 to 0.13, *P* = 5.38E-04) with a proportion of 14.44%.

**Table 1:** The mediation effect of TyG index and AS via plasma metabolites

Mediator	Effect A	Effect B	Mediation effect	*P*	Mediated proportion (%)
	β (95% CI)	β (95% CI)	β (95% CI)		
1-arachidonoyl-gpc (20:4n6)	0.36 (0.13 to 0.58)	0.18 (0.14 to 0.23)	0.06 (0.02 to 0.11)	4.38E-03	11.56%
1-stearoyl-2-arachidonoyl-gpc (18:0/20:4)	0.53 (0.30 to 0.75)	0.15 (0.09 to 0.20)	0.08 (0.03 to 0.12)	6.60E-04	13.89%
1-stearoyl-2-arachidonoyl-gpe (18:0/20:4)	0.96 (0.72 to 1.20)	0.12 (0.07 to 0.17)	0.12 (0.06 to 0.17)	6.58E-05	21.09%
1-palmitoyl-2-arachidonoyl-gpe (16:0/20:4)	0.65 (0.41 to 0.89)	0.10 (0.06 to 0.15)	0.07 (0.03 to 0.11)	8.75E-04	12.24%
1-palmitoyl-2-docosahexaenoyl-gpe (16:0/22:6)	0.78 (0.54 to 1.02)	0.11 (0.05 to 0.17)	0.09 (0.03 to 0.14)	1.26E-03	15.83%
1-oleoyl-2-arachidonoyl-gpe (18:1/20:4)	0.72 (0.49 to 0.96)	0.11 (0.06 to 0.16)	0.08 (0.03 to 0.13)	5.38E-04	14.44%

The term “Total effect” refers to the overall effect of the TyG index on AS. “Effect A” represents the effect of the TyG index on metabolites, and “Effect B” denotes the effect of metabolites on AS. The “Mediation effect” describes the effect of the TyG index on AS mediated through plasma metabolites. The total effect, Effect A, and Effect B were estimated using the inverse variance weighted (IVW) method, whereas the mediation effect was calculated using the delta method. All statistical tests were two-sided, and a *P* value < 0.05 was considered statistically significant

### Power

Given the current sample size, the proportion of variance explained (PVE) by the instrumental variables for the TyG index and the significant metabolites, and the estimated associations with the outcomes, we calculated both the statistical power and the corresponding effect sizes (β) detectable at 80% power, which are reported in Supplementary Table 18.

## Discussion

In this study, we utilized genetic variants as unbiased proxies for the TyG index and conducted MR analyses to explore potential causal relationships between the TyG index, metabolites, and AS risk. The evidence we observed suggests that the genetically predicted TyG index has a positive association with the risk of AS. When employing different MR methods, since these can effectively estimate the causal relationship between exposure and outcomes under their respective hypotheses, it is unlikely that our results are caused by horizontal pleiotropy. Our analysis results are largely robust. Additionally, we conducted a mediation analysis to assess potential mediating effects. Evidence indicates that the impact of the genetically predicted TyG index on AS risk is partially mediated through changes in these metabolites, although the indirect effects are less than the total effects.

To date, although the epidemiological literature examining the association between the TyG index and aortic valve stenosis remains limited, existing observational studies have consistently reported a positive relationship [48]. Specifically, univariate and multivariate logistic regression analyses have demonstrated that a higher TyG index is associated with an increased likelihood of aortic valve calcification (OR [95% CI]: 1.743 [1.036–2.933]) and severe aortic valve calcification (OR [95% CI]: 1.608 [1.143–2.262]). In line with these observations, our MR analysis provides genetic evidence supporting a causal relationship, with the IVW estimate indicating that each one–standard deviation increase in the TyG index is associated with an approximately 70% higher risk of aortic valve stenosis. Although the precise mechanisms linking the TyG index to aortic valve stenosis require confirmation through additional molecular studies, the established correlation between the TyG index and insulin resistance implies that insulin resistance may be a significant driving factor in this association [5]. Insulin resistance (IR) markedly elevates metabolic risk and is strongly associated with several metabolic disorders, including impaired glucose and lipid metabolism [49]. Insulin activates numerous intracellular phosphorylation cascades that enhance aspects of metabolism and cellular processes such as protein synthesis, proliferation, and apoptosis [50]. Valve interstitial cells (VIC) display characteristic components of insulin signaling and develop insulin resistance in conditions of hyperinsulinemia and hyperglycemia—states characterized by disrupted glucose metabolism. Analysis of cultured VIC and aortic valve tissues has revealed remodeling and degenerative alterations in the extracellular matrix [51]. Despite this, up to now, the mechanism of action between insulin resistance and aortic valve stenosis is not yet fully understood, and it remains an important area for future research.

We conducted a two-step MR mediation analysis and found that the impact of the TyG index on AS risk is partially mediated by metabolites, with a relatively modest mediated proportion. Our study indicates that under multiple testing level, 15 distinct metabolites are associated with aortic valve stenosis. Of these, six metabolites serve as mediators in the relationship between the TyG index and aortic valve stenosis. All six metabolites are components of the Lipid super pathway. Specifically, 1-arachidonoyl-gpc (20:4n6) is part of the Lysophospholipid sub-pathway. 1-stearoyl-2-arachidonoyl-gpc (18:0/20:4) is part of the Phosphatidylcholine (PC) sub-pathway. The others—1-stearoyl-2-arachidonoyl-gpe (18:0/20:4), 1-palmitoyl-2-arachidonoyl-gpe (16:0/20:4), 1-palmitoyl-2-docosahexaenoyl-gpe (16:0/22:6), and 1-oleoyl-2-arachidonoyl-gpe (18:1/20:4)—are all classified under the Phosphatidylethanolamine (PE) sub-pathway. In recent years, various metabolomics studies have identified significant metabolic distinctions between patients with AS and healthy individuals. Surendran et al. investigated the tissue-specific metabolic profiles in patients at different stages of calcific aortic valve stenosis (CAS), ranging from mild to severe CAS [52]. Their findings suggested that pathways involved in lipid metabolism and biosynthesis are predominantly associated with the severity of CAS. The metabolic species most significantly correlated included phosphatidylcholine (PC), lysophosphatidylcholine (LysoPC), lysophosphatidylethanolamine (LysoPE), lysophosphatidic acid (LysoPA), and monoacylglyceride (MG). These metabolites were found to have either positive or negative correlations with each other. Specifically, LysoPE, monoacylglyceride (MG), and LysoPA, along with their metabolic species, showed the strongest associations with CAS severity [52]. Furthermore, a study integrating proteomics and metabolomics identified a suite of proteins and metabolites linked to coagulation, inflammation, immune response, ischemia, and lipid metabolism as potential biomarkers that differentiate between calcific aortic valve stenosis and aortic valve regurgitation [53]. Van Driel BO and colleagues demonstrated that the top 30 metabolic features distinctly distinguish AS patients from healthy controls [54]. These features include 17 metabolites related to nitric oxide metabolism, eight associated with fatty acids and phytanic acid, and four steroids and their derivatives. Interestingly, the levels of antioxidant metabolites, nitric oxide metabolism metabolites, and steroids involved in inflammatory pathways normalized to those of healthy individuals four months post-aortic valve replacement (AVR). These changes might indicate their role in exacerbating valve pathologies or suggest adaptive mechanisms to shield the heart or body from the effects of cardiac insufficiency [54]. Furthermore, Al Hageh C and colleagues analyzed the plasma and urine metabolic profiles of AS patients in comparison to healthy controls, successfully identifying biomarkers that distinctly differentiate between these groups. These results underscore the significance of metabolomics in detecting potential disease-related biomarkers and lay a crucial foundation for advancing research into the early diagnosis of AS [55]. In addition, Al-Sulaiti H and colleagues observed that in non-obese participants, insulin resistance and Type 2 Diabetes (T2D) correlate with changes in choline metabolism. These changes include decreased levels of choline and trimethylamine N-oxide, along with increased levels of phosphatidylcholines and phosphatidylethanolamines, as well as their degradation products [56].

Most of the significant metabolites identified in our study fall within the lipid super-pathway, spanning phosphatidylcholine (PC), phosphatidylethanolamine (PE), lysophospholipids, phosphatidylinositol (PI), and diacylglycerols, with homocitrulline representing an amino acid–related pathway (urea cycle; arginine and proline metabolism). Consistent with our findings, recent metabolomics studies have reported substantial metabolic differences between patients with aortic stenosis (AS) / calcific aortic valve disease (CAVD) and controls, with lipid metabolism emerging as a prominent altered domain. For example, Surendran et al. profiled human calcified aortic valves across disease stages and identified significant perturbations in multiple lipid classes and metabolic pathways during progression [52]. In addition, an integrated proteomic–metabolomic analysis of calcific aortic valve disease further highlighted pathways related to inflammation and lipid metabolism as key differentiators between CAVD and controls [57].

Mechanistically, glycerophospholipids (PC/PE/PI) and lysophospholipids are not only structural membrane components but also reservoirs of polyunsaturated fatty acids that can be mobilized to generate bioactive lipid mediators, thereby amplifying inflammatory signaling and promoting osteogenic remodeling of valvular interstitial cells. Moreover, oxidized phospholipids have been implicated in the presence, incidence, and progression of aortic valve calcification and established AS, providing additional support for an oxidative lipid–driven calcification axis in CAVD [58]. Diacylglycerols, also observed among our significant metabolites, are bioactive lipid second messengers involved in metabolic and inflammatory signaling networks, supporting the plausibility that DAG-related dysregulation may contribute to disease biology [59].

Beyond lipid pathways, homocitrulline is a well-recognized marker of protein carbamylation and has been associated with adverse cardiovascular outcomes in recent clinical studies [60]. As a characteristic carbamylation-derived product, homocitrulline reflects enhanced protein carbamylation activity, a process that has also been linked to cardiovascular calcification. Accumulating evidence suggests that carbamylated proteins may promote calcification-related phenotypes through mechanisms involving oxidative stress and vascular dysfunction [61, 62]. Collectively, these prior findings support a working hypothesis that the lipid (PC/PE/PI/lysophospholipid/diacylglycerol) and carbamylation-related pathways highlighted by our results may influence aortic stenosis through interconnected mechanisms involving inflammatory lipid signaling, oxidative stress, and calcific remodeling.

Taken together, our findings support a coherent biological framework linking insulin resistance to the development of aortic valve stenosis. An elevated TyG index reflects systemic insulin resistance, which is accompanied by widespread metabolic disturbances, including chronic low-grade inflammation, dyslipidemia, and altered lipid signaling. These metabolic changes are known to reshape the circulating lipid milieu and promote the accumulation of pro-inflammatory lipid species. In our study, genetically predicted increases in the TyG index were associated with changes in multiple lipid metabolites, particularly glycerophospholipids enriched in polyunsaturated fatty acids, which have been implicated in inflammatory signaling and calcification-related pathways. Such lipid alterations may enhance inflammatory activation, oxidative stress, and osteogenic differentiation of valvular interstitial cells, thereby facilitating fibro-calcific remodeling of the aortic valve. Collectively, these observations suggest that insulin resistance may contribute to aortic valve stenosis through metabolite-mediated pathways linking metabolic dysregulation, lipid-driven inflammation, and valvular calcification.

MR studies offer numerous advantages over Randomized Controlled Trials (RCTs). They are typically more cost-effective, convenient, and swift as they leverage existing large-scale Genome-Wide Association Study (GWAS) data. MR studies adeptly investigate potential causal relationships between modifiable risk factors and rare diseases, which, in the context of RCTs, necessitate large sample sizes and prolonged tracking to gather adequate endpoints. Achieving long-term adherence in RCTs can be challenging, and associated costs tend to escalate over time. Consequently, MR methodologies are particularly useful for assessing and contrasting the TyG index with its correlation to AS within identical cohorts. Moreover, since genetic variations are predetermined at conception, the outcomes of MR studies provide insights into the lifelong impacts on risk factors, thus addressing several limitations inherent in traditional observational studies and RCTs. By restricting the study population to individuals of European descent, this approach effectively minimizes bias due to population stratification.

However, our findings should be interpreted with certain limitations in mind. Firstly, to minimize potential confounding due to population stratification, our analysis was confined to participants of European ancestry. Although the risk of population overlap is minimal, the generalizability of our findings to other populations remains uncertain, underscoring the necessity for further research in non-European cohorts. Secondly, the selection criteria for instrumental variables were extremely stringent, incorporating P-value corrections, and the sample size for the GWAS data source for plasma metabolites was relatively small. To uphold robustness, as required by some methods, we had no choice but to exclude certain metabolites. Concurrently, the unidentified molecular structures of some metabolites challenge our understanding. With the availability of data from more participants, replicating these findings using larger and more potent GWAS for plasma metabolites will allow us to draw more definite conclusions. Moreover, it should be acknowledged that the lack of information on the severity of aortic valve stenosis may be a limiting factor. Besides, Because the TyG index instruments were derived from externally published GWAS, in which individuals with diabetes or dyslipidemia were excluded, and alternative GWAS including these populations were unavailable, residual selection bias cannot be completely ruled out. In addition, because the TyG index instruments were derived from externally published GWAS that excluded individuals with diabetes or dyslipidemia, and alternative GWAS including these populations were unavailable, residual selection bias cannot be completely ruled out. Finally, despite our meticulous efforts to satisfy statistical criteria for homogeneity and absent horizontal pleiotropy, we could not entirely account for potential biological pleiotropy due to the intricate genetic and environmental factors related to the TyG index, metabolites, and AS.

In conclusion, our MR analysis has confirmed a causal relationship between genetically determined levels and the risk of AS. Robust genetic evidence indicates a pathogenic connection between a higher TyG index and an increased risk of AS. Additionally, certain metabolites modestly mediate this effect. Further comprehensive studies are required to determine whether the concentrations of these metabolites in the plasma of individuals with a high TyG index can be modulated through pharmacological interventions or other methods, potentially influencing the progression of AS.

## Supplementary Information

Supplementary Material 1

## Data Availability

All data generated or analyzed during this study are included in this published article and its supplementary information files. Further inquiries should be directed to the corresponding author.

## Abbreviations

MR: Mendelian randomization
SNPs: Single nucleotide polymorphisms
AS: Calcific aortic stenosis
PVE: Proportion of variance explained
SD: Standard deviation
CI: Confidence interval
OR: Odds ratio
TyG: Triglyceride-glucose
GWAS: Genome-wide association studies
